# Antioxidant Potential of Extracts Obtained from Macro- (*Ascophyllum nodosum*, *Fucus vesiculosus* and *Bifurcaria bifurcata*) and Micro-Algae (*Chlorella vulgaris* and *Spirulina platensis*) Assisted by Ultrasound

**DOI:** 10.3390/medicines5020033

**Published:** 2018-04-10

**Authors:** Rubén Agregán, Paulo E. S. Munekata, Daniel Franco, Javier Carballo, Francisco J. Barba, José M. Lorenzo

**Affiliations:** 1 Centro Tecnológico de la Carne de Galicia, Adva. Galicia No. 4, Parque Tecnológico de Galicia, San Cibrao das Viñas, 32900 Ourense, Spain; rubenagregan@ceteca.net (R.A.); danielfranco@ceteca.net (D.F.); 2 Department of Food Engineering, Faculty of Animal Science and Food Engineering, University of São Paulo, 225 Duque de Caxias Norte Ave, Jardim Elite, Pirassununga 13635-900, São Paulo, Brazil; pmunekata@gmail.com; 3 Area de Tecnologia de los Alimentos, Facultad de Ciencias de Ourense, Universidad de Vigo, 32004 Ourense, Spain; carbatec@uvigo.es; 4 Nutrition and Food Science Area, Preventive Medicine and Public Health, Food Science, Toxicology and Forensic Medicine Department, Universitat de València, Avda. Vicent Andrés Estellés, s/n, Burjassot, 46100 València, Spain; francisco.barba@uv.es

**Keywords:** macroalgae, microalgae, extraction yield, total phenolic content, antioxidant activity

## Abstract

**Background:** Natural antioxidants, which can replace synthetic ones due to their potential implications for health problems in children, have gained significant popularity. Therefore, the antioxidant potential of extracts obtained from three brown macroalgae (*Ascophyllum nodosum*, *Fucus vesiculosus* and *Bifurcaria bifurcata*) and two microalgae (*Chlorella vulgaris* and *Spirulina platensis*) using ultrasound-extraction as an innovative and green approach was evaluated. **Methods:** Algal extracts were obtained by ultrasound-assisted extraction using water/ethanol (50:50, *v*:*v*) as the extraction solvent. The different extracts were compared based on their antioxidant potential, measuring the extraction yield, the total phenolic content (TPC) and the antioxidant activity. **Results:** Extracts from *Ascophyllum nodosum* (AN) and *Bifurcaria bifurcata* (BB) showed the highest antioxidant potential compared to the rest of the samples. In particular, BB extract presented the highest extraction (35.85 g extract/100 g dry weight (DW)) and total phenolic compounds (TPC) (5.74 g phloroglucinol equivalents (PGE)/100 g DW) yields. Regarding the antioxidant activity, macroalgae showed again higher values than microalgae. BB extract had the highest antioxidant activity in the ORAC, DPPH and FRAP assays, with 556.20, 144.65 and 66.50 µmol Trolox equivalents (TE)/g DW, respectively. In addition, a correlation among the antioxidant activity and the TPC was noted. **Conclusions:** Within the obtained extracts, macroalgae, and in particular BB, are more suitable to be used as sources of phenolic antioxidants to be included in products for human consumption. The relatively low antioxidant potential, in terms of polyphenols, of the microalgae extracts studied in the present work makes them useless for possible industrial applications compared to macroalgae, although further in vivo studies evaluating the real impact of antioxidants from both macro- and micro-algae at the cellular level should be conducted.

## 1. Introduction

Free radicals may produce damage to lipids, proteins, cell membranes and nucleic acids, thus promoting the development of noncommunicable diseases [1]. Therefore, an increased interest in natural antioxidants to fight against free radicals has been shown. Consumers are particularly interested in natural antioxidants rather than synthetics [2] due to the problems of toxicity and carcinogenic effects that these may cause [3,4].

Brown seaweed species are rich in antioxidant polyphenols (from 1–14% dry solid), *Ascophyllum* and *Fucus* being two genera with the highest content [5]. Polyphenols are reported to possess benefits for heath, such as anticancer, antimicrobial, anti-inflammatory and antidiabetic activities [6]. Brown algae are rich in phlorotannins [5], polyphenols with multiple phenolic groups, which provide good antioxidant activities [7]. These compounds are exclusively from brown algae species [8,9].

In the same way as macroalgae, microalgae could represent an important source of antioxidant compounds [10,11]. They are a rich source of natural pigments with antioxidant properties, such as chlorophylls and carotenoids, thus giving an added value from a commercial point of view [12]. Moreover, their biodiversity, ease of cultivation and modulation of growth conditions has resulted in microalgae becoming among the most important resources in nature having antioxidant properties [13].

Conventional extraction has been traditionally used to recover antioxidants from algae. However, this presents some important drawbacks such long extraction time, high cost and degradation of product quality. In addition, the use of organic solvents as extractive compounds should be minimized since they may be harmful from a health and environmental point of view [14,15]. For this reason, non-conventional extraction methods were employed to recover bioactives in food and pharmaceutical applications, providing satisfactory results, such as reducing process time and cost and increasing yield [16,17]. Ultrasound-assisted extraction (UAE) is an innovative extraction approach, which is based on sound wave migration, thus promoting cavitation phenomena and leading to a disruption of cell walls and the subsequent release of intracellular compounds [18]. This extraction method offers many advantages, such as a better solvent penetration into cellular material, higher product yields and reproducibility, lower solvent consumption and higher processing throughput compared to the conventional extraction methods [19]. Actually, UAE is very often used in the extraction of natural antioxidant compounds [20]. It has been employed in the extraction of antioxidant compounds from seaweeds with satisfactory results [21,22,23]. Therefore, the aim of this work was to evaluate the potential use of *Ascophyllum nodosum* (AN), *Fucus vesiculosus* (FV) and *Bifurcaria bifurcata* (BB) macroalgae extracts and *Chlorella vulgaris* (CV) and *Spirulina platensis* (SP) microalgae extracts as antioxidants for the possible application in products intended to be used for human consumption. For this purpose, UAE will be used as the extraction technology to recover the antioxidant bioactive compounds.

## 2. Materials and Methods

### 2.1. Algal Material

Brown macroalgae, *Ascophyllum nodosum* (AN), *Fucus vesiculosus* (FV) and *Bifurcaria bifurcata* (BB) (Chromista, Ochrophyta, Phaeophyceae, Fucales), were purchased to Portomuiños company (A Coruña, Spain). The collection was carried out in the Atlantic Ocean near the Camariñas area (A Coruña, Spain). Microalgae, *Chlorella vulgaris* (CV) (Plantae, Chlorophyta, Trebouxiophyceae, Chlorellales) and *Spirulina platensis* (SP) (Eubacteria, Cyanobacteria, Cyanophyceae, Spirulinales) were provided by AlgaEnergy (Madrid, Spain). Macroalgae samples were ground obtaining particles lower than 0.8 mm, by a conventional mincer. Then, algae were stored under vacuum (75%) at −20 °C until further use.

### 2.2. Obtaining Extracts from Macroalgae and Microalgae by UAE Method

Each of the algae (5 g) was extracted using a mixture consisting of water/ethanol (50:50, *v*:*v*) (50 mL) in glass bottles. The extraction was performed in an ultrasonic bath (Branson ultrasonic M3800-E, Dietzenbach, Germany) at room temperature for 30 min. After extraction, the solvent was separated from the alga by centrifuging at 3000× *g* for 10 min at 4 °C and filtering with filter paper using a vacuum to remove the algal material. The supernatant was stored at −20 °C until analysis. Extraction yield, total phenolic content (TPC) and antioxidant activity (ORAC, ABTS, DPPH and FRAP assays) were evaluated for the obtained extracts, as described below. All experiments were performed in duplicate.

### 2.3. Measurement of the Extraction Yield

Five milliliters of each extract were taken and evaporated in a drying oven at 100 °C overnight. The weight of the final residue was used to calculate the extraction yield by gravimetry. Results were expressed as g extract/100 g dry weight of algae (DW). The DW of each alga was calculated subtracting its moisture content to total weight of the algae. Moisture content was measured according to the method previously reported in the ISO recommendation [24].

### 2.4. Determination of the Total Phenolic Compounds

TPC determination was based on a method described by Medina-Remón et al. [25]. Fifteen microliters of sample were mixed with 170 µL of Milli-Q water, adding later 12 µL of Folin-Ciocalteu reagent and 30 µL of sodium carbonate. The mixtures were incubated for 1 h at room temperature under darkness. Once the reaction was over, 73 µL of Milli-Q water were added with a multichannel pipette. The absorbance measurement was performed at 765 nm. TPC was expressed as g of phloroglucinol equivalents (PGE)/100 g extract and as g PGE/100 g DW.

### 2.5. Determination of the Antioxidant Activity

#### 2.5.1. Oxygen Radical Absorbance Capacity Assay

The original method of Ou et al. [26] modified by Dávalos et al. [27] was used. The reaction was carried out in 75 mM phosphate buffer (pH 7.4), 200 µL being the final reaction mixture. The mixture with antioxidant (20 µL) and fluorescein (120 mL; 70 nM final concentration) was pre-incubated for 15 min at 37 °C. AAPH (2,20-azobis (2-methylpropionamidine) dihydrochloride) solution (60 mL; 12 mM, final concentration) was added rapidly using a multichannel pipette. The plate was immediately placed in the reader, and the fluorescence was recorded every minute for 120 min (excitation wavelength 485 nm, emission wavelength 520 nm). Samples were stirred prior to each reading. Eight calibration solutions using Trolox as the antioxidant were used in each assay, and phosphate buffer was used as blank. Results were calculated on the basis of the differences in areas under the fluorescein decay curve between the blank and the sample and were expressed as µmol Trolox equivalents (TE)/g DW.

#### 2.5.2. ABTS Radical Cation Decolorization Assay

The method of Re et al. [28] was adapted to the use of a plate reader. ABTS^•+^ was produced by mixing 7 mM ABTS stock solution with 2.45 mM potassium persulfate (final concentration) leaving the mixture in the dark at room temperature for 12–16 h before use. In the following step, ABTS^•+^ solution was diluted with PBS (pH 7.4) to get an absorbance of 0.70 at 734 nm, being equilibrated at 30 °C. The working solution of ABTS^•+^ (sample: ABTS solution relation, 1:100) was added to an aliquot of each sample (with appropriate dilution). The decrease in absorbance was measured after 6 min at 734 nm in a microplate spectrophotometer reader. Trolox was used as the reference standard, and the results were expressed as µmol TE/g DW.

#### 2.5.3. DPPH Radical Scavenging Assay

The DPPH^•^ scavenging method was performed with some modifications according to the procedure previously described by Brand-Williams et al. [29]. Five microliters of samples (previously diluted) were added to 195 µL of DPPH solution (6 × 10^−5^ M in methanol) in 96-well plates. The mixture was lightly shaken and left at room temperature for 30 min. Then, the absorbance at 515 nm was measured against methanol using a microplate reader. The DPPH^•^ scavenging activity of extracts was determined using the standard curve of Trolox and expressed as µmol TE/g DW.

#### 2.5.4. Ferric Reducing Antioxidant Power 

Ferric reducing antioxidant power was determined using the method described by Benzie and Strain [30]. The FRAP reagent was freshly prepared from 300 mM acetate buffer, pH 3.6, 10 mM 2,4,6-tripyridyl-s-triazine (TPTZ) made up in 40 mM HCl and 20 mM FeCl_3_·6H_2_O solution. The three solutions were mixed together in the ratio of 10:1:1 (*v*:*v*:*v*). Three-hundred microliters of freshly-prepared FRAP reagent were mixed with 10 µL of properly diluted samples and 30 µL of distilled water in 96-well plates. The mixture was heated at 37 °C and left at this temperature during the reaction. After 8 min, the absorbance was measured using a microplate reader at 593 nm against reagent blank. The FRAP value was calculated and expressed as µmol TE/g DW based on a calibration curve plotted using Trolox as the standard.

## 3. Results and Discussion

### 3.1. Extraction Yield

Extraction yields from different algae are presented in Table 1. AN and BB macroalgae showed higher extraction yields compared to FV and microalgae species. Specifically, BB achieved the highest value (35.85 and 35.85 g extract/100 g DW). CV and SP microalgae presented lower extraction yields than macroalgae, SP being the one that had the lowest value (4.56 and 4.24 g extract/100 g DW). Farvin and Jacbsen [31] also found a lower extraction yield for FV compared to most of the algae used in their study when using water as the solvent. They attributed this fact to the important viscosity found in these extracts, which made the filtering process through filter paper difficult. On the other hand, other authors also reported significant differences in extraction yield among several seaweed species using different extraction solvents, such as water, ethanol or diethyl ether [31,32]. Matanjun et al. [32] found a positive correlation among yield and solvent polarity when extracting antioxidant bioactive compounds from eight seaweed species from north Borneo. At the same time, they found differences between algae using the solvents separately. According to them, the variation in the yields from several extracts may be due to the different polarities of the compounds found in plants [31]. Other factors that can explain the modifications in extraction yields are (i) the chemical composition of the raw material and (ii) the polarity of the solvents used [33]. For instance, different solvents, such as ethanol, water or aqueous solutions of organic solvents, were tested to obtain the optimal solvent for improving extraction yields, obtaining the best results when ethanol was used [31,34]. Thus, taking into account the extraction solvent used (ethanol:water 50:50, *v*:*v*), it could be said that BB and AN macroalgae are richer in polar compounds than FV and both microalgae species. The observed lower content of polar compounds found in CV and SP and their extracts may be related to the amount of carotenoids present in microalgae. Microalgae are a good source of carotenoids [35,36]. For instance, *Chlorella vulgaris* is reported to contain high amounts of carotenoids, such as lutein and β-carotene [37,38]. As is well known, carotenoids are lipophilic compounds [39], making their extraction difficult with polar solvents, such as ethanol, water or mixtures of both, as in our study. Therefore, the extraction yields from CV and SP microalgae were probably affected by the use of water/ethanol (50:50, *v*:*v*) as the extraction solvent.

### 3.2. Total Phenolic Content 

TPCs from the different algal extracts are presented in Table 1. As can be seen in the table, AN and BB macroalgae showed higher TPC contents compared to FV and both microalgae. BB presented the highest content (5.74 and 5.74 g PGE/100 g DW). It should be noted that microalgae presented lower TPCs than macroalgae, SP being the one that reached the lowest content (0.20 and 0.19 g PGE/100 g DW). The differences (*p* < 0.05) observed between TPC in macro- and micro-algae could be due to the high content of our three macroalgae in phlorotannins. As already mentioned above, brown algae genera are rich in these compounds [6], composed of units of phloroglucinol joined to form polymers [31]. In addition, phlorotannins are bi-polar in nature [40]; therefore, they are soluble in polar solvents such as in the aqueous solution at 50% ethanol used in this study, ensuring their presence in the extract and strengthening our hypothesis. These compounds could help algae in their struggle against oxidative stress, as well as participate in the defense against grazers such as marine herbivores thanks to their plasticity [41].

Chew et al. [42] found a much higher TPC content in the *Padina antillarum* brown alga than in the *Caulerpa racemosa* green alga and in the *Kappaphycus alvarezii* red alga. They also attributed this fact to the phlorotannin presence in the brown algae. On the other hand, we did not only find that macroalgae had different TPCs (*p* < 0.05) than microalgae, but that all algae studied showed different total content (*p* < 0.05) in phenolic compounds. Connan et al. [43] reported that external-environmental factors, such as light, depth or salinity, and intrinsic factors, such as age or length, may affect the phenolic metabolic expressions of algae, generating great differences in the phenolic content [43,44].

Observing the extraction yield data, a correlation between extraction yields and TPC was noted, since the highest extraction yield values corresponded to the highest TPC values, the order being increased in the following way: BB > AN > FV > CV > SP. This correlation is related to the percentage of TPC in the extracts. Moreover, this percentage, as can be expected, was very similar for both macroalgae (16–20 g PGE/100 g extract) and for microalgae (4.3–4.7 g PGE/100 g extract).

The differences observed for TPC in the different algae samples could be attributed to different factors, such as the period of the year or area in which they are collected. Hold and Kraan [6] reported that polyphenol content showed a correlation with the reproductive state of algae along time. Polyphenol content in *Ascophyllum nodosum* is minimum during May, the month with maximal fruit body shedding, and maximum in winter. However, March is the month in which the minimum of TPC was observed for *Fucus vesiculosus*, just before the period of maximum fertility [45]. On the other hand, *Porphyra umbilicalis* alga was affected by sun exposure and emersion. The authors noted that seaweeds exposed to air and water during the summer contained higher amounts of antioxidants than submerged seaweeds, submersion being a natural barrier for seaweeds against environmental stresses [46].

### 3.3. Antioxidant Activity

Antioxidant activities of the different algae extracts are presented in Table 1. Macroalgae showed higher antioxidant activities than microalgae for all the assays. As discussed above, brown algae are rich in phlorotannins showing high antioxidant activities. Ahn et al. [47] reported an interesting antioxidant capacity from three phlorotannins extracted from *Ecklonia cava* brown alga. Within macroalgae, BB had the highest values (537.38 and 575.02, 143.04 and 146.27, 67.40 and 65.60 µmol TE/g DW in the ORAC, DPPH and FRAP assays, respectively) and FV the lowest. This indicates that the BB algae are richer in compounds capable of scavenging free radicals. As expected, a positive correlation between TPC and antioxidant activity was noted. Thus, BB, which showed the highest TPC, also presented the highest antioxidant activity in almost all assays, followed by FV, AN and microalgae. The same correlation was reported by other authors after using the DPPH radical scavenging assay [42]. They found that when TPC content was higher, the IC_50_ decreased. According to these authors, the polyphenols present in seaweeds have the ability to scavenge free radicals. By using the DPPH assay, it was also found that seaweed extracts containing high levels of phenolic compounds also displayed potent antioxidant activities [31]. This correlation may mean that polyphenols are the compounds that contribute most to the antioxidant activity of our extracts. Other authors also came to the same conclusion with their extracts [31,42]. Focusing on microalgae, there are studies in which the results obtained were contradictory. On the one hand, it was found that the antioxidant capacity of microalgae is partly caused by polyphenols [48]. However, other authors did not find any correlation between the phenolic content and the antioxidant capacity of ethanolic extracts resulting from nine microalgae strains [49].

The determination of phenolic compounds as individual molecules in the extracts is, therefore, of great importance for radical scavenging activity [31]. This activity is also dependent on the structure of the compounds, as well as the amount and location of the hydroxyl groups in them [29]. For example, some studies found that caffeic acid, which has two hydroxyl groups, is a compound with greater antiradical activity than coumaric acid, a homolog of caffeic acid, but with only one hydroxyl group [29]. Therefore, the different phenolic combinations of the compounds will have an impact on the antioxidant activity of these [31].

## 4. Conclusions

Extraction yield, TPC and antioxidant activity from macroalgae extracts obtained by the UAE method using water/ethanol (50:50, *v*:*v*) as the extraction solvent turned out to be higher than microalgae extracts obtained in the same way, meaning that macro-algae extracts, specially BB extract, are more suitable to be used as possible high-polyphenol antioxidants in products to be used for human consumption. The combined use of the aforementioned extraction method and solvent was inefficient to obtain microalgae extracts with good extraction yields and antioxidant potential. Therefore, these extracts obtained in this way are not interesting for possible industrial applications, presenting drawbacks, such as the cost or the amount that must be added to the product. Taking into account the data obtained, if additional research is carried out, it should be focused on BB algae extract. In addition, it would be interesting to assess the real impact of antioxidants from both macro- and micro-algae at a cellular level in further research.

## Figures and Tables

**Table 1:** Extraction yields, TPCs and antioxidant activities from AN, FV, BB, CV and SP algae extracts obtained by the UAE method using water/ethanol (50:50, *v*:*v*) as the extraction solvent.

Algae	Extraction Yield		TPC				Antioxidant Activities							
							ORAC		ABTS		DPPH		FRAP	
	g/100 g DW		g PGE/100 g Extract		g PGE/100 g DW		µmol TE/g DW							
AN	25.86	25.86	18	18	4.66	4.66	297.19	300.29	565.66	542.38	50.69	50.69	4.66	4.40
FV	9.58	9.80	20	20	1.92	1.96	155.41	154.26	200.60	208.46	26.72	27.15	3.45	3.82
BB	35.85	35.85	16	16	5.74	5.74	537.38	575.02	537.74	549.21	143.04	146.27	67.40	65.60
CV	7.78	7.14	4.5	4.7	0.35	0.34	33.07	29.35	15.64	14.64	0.86	0.79	0.62	0.62
SP	4.56	4.24	4.3	4.6	0.20	0.19	12.30	12.12	6.74	6.53	1.00	0.89	1.00	1.02

Algae: AN, *Ascophyllum nodosum*; FV, *Fucus vesiculosus*; BB, *Bifurcaria bifurcata*; CV, *Chlorella vulgaris*; SP, *Spirulina platensis*. UAE, ultrasound–assisted extraction. TPC, total phenolic content. DW, dry weight of alga. PGE, phloroglucinol equivalents. TE, Trolox equivalents.
